# Lactate: A Theranostic Biomarker for Metabolic Psychiatry?

**DOI:** 10.3390/antiox12091656

**Published:** 2023-08-22

**Authors:** Edward Caddye, Julien Pineau, Joshua Reyniers, Itamar Ronen, Alessandro Colasanti

**Affiliations:** 1 Clinical Imaging Sciences Centre, Brighton and Sussex Medical School, University of Sussex, Falmer BN1 9RR, UK; 2 Department of Clinical Neuroscience, Brighton and Sussex Medical School, University of Sussex, Falmer BN1 9RR, UK; 3 Independent Researcher, Florianópolis 88062-300, Brazil; 4 School of Life Sciences, University of Sussex, Falmer BN1 9RR, UK

**Keywords:** brain, imaging, lactate, mitochondria, metabolic psychiatry, neurometabolism, redox

## Abstract

Alterations in neurometabolism and mitochondria are implicated in the pathophysiology of psychiatric conditions such as mood disorders and schizophrenia. Thus, developing objective biomarkers related to brain mitochondrial function is crucial for the development of interventions, such as central nervous system penetrating agents that target brain health. Lactate, a major circulatory fuel source that can be produced and utilized by the brain and body, is presented as a theranostic biomarker for neurometabolic dysfunction in psychiatric conditions. This concept is based on three key properties of lactate that make it an intriguing metabolic intermediate with implications for this field: Firstly, the lactate response to various stimuli, including physiological or psychological stress, represents a quantifiable and dynamic marker that reflects metabolic and mitochondrial health. Second, lactate concentration in the brain is tightly regulated according to the sleep–wake cycle, the dysregulation of which is implicated in both metabolic and mood disorders. Third, lactate universally integrates arousal behaviours, pH, cellular metabolism, redox states, oxidative stress, and inflammation, and can signal and encode this information via intra- and extracellular pathways in the brain. In this review, we expand on the above properties of lactate and discuss the methodological developments and rationale for the use of functional magnetic resonance spectroscopy for in vivo monitoring of brain lactate. We conclude that accurate and dynamic assessment of brain lactate responses might contribute to the development of novel and personalized therapies that improve mitochondrial health in psychiatric disorders and other conditions associated with neurometabolic dysfunction.

## 1. Introduction

Metabolic psychiatry is an emerging field which aims to improve outcomes amongst those suffering with psychiatric disorders by therapeutically targeting metabolism. This subspecialty is grounded in the bidirectional association of metabolic (e.g., obesity and type 2 diabetes) and mood and affective disorders, such as bipolar disorder (BPD), major depressive disorder (MDD), and schizophrenia (SCZ) [1].

Cellular metabolic homeostasis is crucially regulated by mitochondria, bi-genomic organelles which integrate environmental signals and coordinate cellular fate, circadian rhythms, inflammation, and immunity [2]. The role of mitochondria in human health and disease is increasingly recognised [3], and it is particularly relevant for understanding the pathophysiology of brain disorders. In the central nervous system (CNS), their function is universal and complex due to the exceptional energetic demands that is required by higher-order brain activity [4]. In rare disorders caused by mutations in genes that encode for mitochondrial proteins, neurological impairment is common, and there is an elevated risk of developing comorbid psychiatric disorders [5]. The complexity is amplified by the fact that mitochondria accumulate and express mitochondrial (mt)DNA mutations throughout their lifespan, are involved in brain ageing and neurogenerative processes [6], and respond to psychological stress [7].

### 1.1. The Role of Mitochondria in the Pathogenesis of Psychiatric Disorders

The role of mitochondria and neurometabolism in the aetiopathophysiology of mood and affective disorders has been reviewed extensively [8,9,10]. Pre-clinical, post-mortem, cerebrospinal fluid (CSF) sampling, and imaging studies have yielded valuable insight in MDD, BPD, and SCZ thus far. Of note, chronic mild stress dissipates membrane potential in mitochondria and damages brain mitochondrial ultrastructure in mice [11]. In a pre-natal stress mouse model of depression, the rate of glycolysis is increased, and oxidative metabolism is reduced [12]. This is consistent with the finding that in depressed adults, there are elevated concentrations of the mitochondrial fuel lactate in the anterior cingulate cortex (ACC) [13] and CSF [14]. In fact, the finding of elevated CSF lactate is consistent across MDD, BPD [15], and SCZ [14]. Despite the nomenclature, the gene Disrupted In Schizophrenia 1 (DISC1) confers risk for all mental illnesses [16]. DISC1 is implicated in astrocytic energy metabolism and regulates glucose uptake and lactate production [17]. In SCZ, there are abnormalities of both glycolytic and oxidative enzyme function [18] and a reduction in axonal mitochondrial density in the ACC [19], which is consistent with in vivo imaging showing a reduced creatine kinase (CK) reaction rate for ATP synthesis and pH [20]. Elevated ACC lactate levels negatively correlate with both general cognition and functional capacity in SCZ [21]. In patients with first episode psychosis, peripheral blood mononuclear cells (PBMCs) have significantly increased protein expression of both lactate dehydrogenase B (LDHB) and glucose-6-phosphate isomerase (GPI), highlighting the need to consider mitochondria and metabolic markers in the brain and the body [22].

In BPD, mitochondrial dysfunction may play a particularly important role. The post-mortem profiling of BPD patients has revealed abnormal mitochondrial morphology [23] and impaired prefrontal cortex (PFC) electron transport chain (ETC) complex assembly, specifically mitochondrial complex 1 (MC1), with increased oxidative damage [24,25]. There is also reduced hippocampal expression of the MC1 subunit gene, NDUFV2 [26]. Changes in mtDNA across several brain regions have been mapped in BPD, with data indicating the vulnerability of different brain regions to energetic demands, for example, with increased and decreased mtDNA levels present in the hippocampus and PFC compared to control post-mortem brains, respectively [27]. Phosphorus magnetic resonance spectroscopic (MRS) studies in BPD patients identified alterations in phosphocreatine [28], creatine kinase reaction rate constant [29], and ATP levels after stimulation [30], whilst proton MRS imaging studies have consistently found elevated brain lactate in BPD (discussed further in the following sections), highlighting the fact that neurometabolic alterations can be detected post-mortem as well as in the living brain of BPD patients [31,32].

Whilst much research has focused on differences between people with these distinct diagnoses and non-affected participants, it would also be important to consider the broader impact of mitochondrial dysfunction on human behaviour and health and transcend the traditional categorical diagnostic criteria and boundaries for psychiatric and neurological diseases. Ideally, mitochondrial dysfunction should be conceptualised and studied through the framework of the six domains of the Research Domain Criteria (RDoC) [33] (negative valence, positive valence, cognitive, social, arousal, and sensorimotor systems) [34]. Either way, there is a clear and unmet need in psychiatry for biomarkers that can be mapped across these domains and linked back to quantitative measures of mitochondrial function.

### 1.2. Potential of Cerebral Lactate as a Neurometabolic Biomarker

Lactate, a major circulatory fuel source that can be produced and utilized by the brain and body, is an excellent candidate to serve as a theranostic biomarker for neurometabolic dysfunction. In sports and exercise medicine, the ability to clear fatty acids and lactate from blood over time is a proxy assessment of systemic oxidative capacity [35], i.e., the efficiency of mitochondrial function at the level of the whole body [36]. Systemic lactate measurements have also been found to inform mechanisms of psychiatric phenomena: early work studying blood lactate dynamics observed that stress precipitated ‘unusual responses at lower stimulus levels’ in those suffering from ‘neurocirculatory asthenia’ (a historical diagnostic category now superseded by elements of anxiety and panic disorder) compared to control participants [37]. Exercising anxious subjects exhibited abnormalities of lactate metabolism that were comparable to those with cardiovascular disease [38,39,40]. This prompted the ‘lactate hypothesis of anxiety’ and the characterisation of exogenous sodium lactate as a panicogenic agent [41]. More recently, lactate has also been purported to have antidepressant properties [42], and the field has received much attention with lactate going from ‘from rags to riches’ [43], once ‘the ugly duckling of metabolism’ [44], now ‘a phoenix risen’ [45]. 

Despite its potential as a biomarker, the peripheral concentration of lactate lacks the ability to adequately reflect mitochondrial function within the brain (and the body), due to the heterogenous distribution of mitochondrial populations between cells, tissues, and organs with different heteroplasmy rates [46]. In fact, it is the tissue specific heteroplasmy rate that may dictate which systems are implicated in mitochondrial disease and influence the profile of neuropsychological symptoms [47]. Despite decades of development and progress in quantitative neurometabolic imaging techniques including positron emission tomography (PET), or magnetic resonance spectroscopy (MRS)-derived measurements of cerebral glucose or oxygen metabolic rate, there is a case to be made for integrating these with tools that have greater specificity for the pathological processes of interest. The MRS techniques enable spatiotemporal resolution of metabolites relevant to bioenergetics, and the characterisation of brain lactate dynamic responses through functional MRS (fMRS) is a promising tool to enable the direct assessment of mitochondrial homeostatic responses to neurometabolic stress [48]. Although measurement of lactate with MRS is challenging due to a low signal-to-noise ratio (SNR) in vivo, we opine that lactate has a key role that enables an integrated and precise approach for appreciating and understanding energy allocation and mitochondrial efficiency within the CNS that goes beyond oxygen and blood flow measurement [49]. Indeed, changes in brain lactate metabolism measured with MRS have already been used to characterise and monitor the response to pharmacological therapy in psychiatric conditions (see Section 4). 

### 1.3. Aim of the Review

In the present review, we hope to drive attention to the potential and under-explored significance of brain lactate as a dynamic biomarker for neurometabolic dysfunction that is relevant to many psychiatric conditions and the RDoC neurobehavioural domains. We will define characteristics of lactate in cells and the brain, outlining the molecular and physiological mechanisms by which it may signal and modulate single neurons, brain nuclei, cognition, and behaviour. We outline how the spatiotemporal regulation of lactate dynamic responses may reflect the state of arousal in the nervous system and consider the implications of these altered lactate dynamics that may occur due to mitochondrial and metabolic dysfunction. 

These observations highlight the possible clinical utility and characterisation of brain lactate metabolism as a potential therapeutic target, which could inform future empirical testing of this hypothesis. If confirmed via rigorous experimental studies through testing the effect of treatment interventions, the characterisation of brain lactate dynamic responses with fMRS could represent an in vivo precision medicine tool to track the effects of novel or pre-existing therapies that target brain metabolism and mitochondrial function, including pharmacological agents but also personalized exercise protocols, dietary interventions, and nutraceuticals.

## 2. Lactate Neurophysiology

Lactate is a three-carbon monocarboxylate that exists predominantly as a negatively charged anion at a physiological pH [50]. It is chiral and can exist as three isomers: DL-lactate, D-lactate, and L-lactate. In the brain, L-lactate (referred to as lactate from now on, unless otherwise specified) is the dominant form [51]. It is produced intracellularly with NAD^+^ via glycolysis and exists in equilibrium with the redox couple pyruvate and NADH [44]. Long thought of as a dead-end waste product produced in response to hypoxia, it is now considered to be the obligatory end-product of glycolysis and a crucial substrate that fuels mitochondrial bioenergetics [43]. Lactate is enriched around mitochondria with concentrations in the range of ~268–1025 µM (<100 µM in the nucleus/cytosol) [52]. Here, it acts as an intermediary between glycolysis and oxidative metabolism, generating adenosine triphosphate (ATP) via pyruvate, or shuttling between other cells, tissues, and organs to fuel the tricarboxylic acid cycle (TCA) or replenish glucose [53]. Lactate is the most abundant carbon metabolite in the blood after glucose, with an average circulating concentration of 1 mM at rest [54,55]. When the rate of systemic uptake is limited by impaired oxidative metabolism, lactate concentrations become elevated, as evidenced by primary mitochondrial disease [56]. Similar observations can be made in obesity and in those with metabolic syndrome (MetS) [57]. Conversely, when the mitochondrial capacity of muscle and other tissues is enhanced by physical training, the clearance of lactate can become more efficient [58,59]. With these two ends of the spectrum in mind, Brooks and San-Millán developed a clinical tool and proxy for measuring systemic mitochondrial health using serial lactate measurements under incremental exercise intensity testing [35]. 

The concentration of lactate in the brain exhibits a robust circadian rhythm [60]. In rodent models, the baseline concentration of 350–420 μM measured with biosensors cycled between −16.2% and +53% [61]. This rhythm was found to be actively regulated by the drainage systems that can clear lactate during natural sleep [62]. Microdialysis data show that cortical lactate is lower during sleep, and lower still during deep sleep [63,64]. These state-dependent changes are controlled both by interstitial clearance mechanisms and an activity-dependent reduction in glycolysis, which can be induced with anaesthesia [65]. To reinforce this relationship, peripheral administration of lactate can trigger wakening [66], and when mice are startled from sleep, lactate rises as much as 50% above baseline [61]. Brain lactate concentration is highly correlated with state-dependent changes in arousal and the concentration of norepinephrine (NE), and it oscillates across the sleep–wake cycle, which is altered by circadian disruption and aging [60,67]. Although glycolytic rates are lower during sleep than during wakefulness [68], the brain remains a net exporter of lactate, at least in healthy individuals at rest [69]. 

Neurons and astrocytes both carry out glycolysis and export lactate [70]. Astrocytes contain stores of glycogen and exhibit a more glycolytic phenotype and hence produce the majority of cerebral lactate [71,72,73]. Somatosensory stimulation triggers the activation of neurons and increases the local concentration of lactate in the brain, linking transmission and neurometabolism [74,75]. Still somewhat debated, astrocytes provide metabolic support for working neurons, a concept underpinned by the astrocytic-neuronal lactate shuttle (ANLS) [76,77]. Of particular relevance to psychiatry with the RDoC domains in mind, data implicate the ANLS in arousal [78], stress responses [79], memory formation [80], and memory processing [81]. Similarly, chronic stress depletes astrocytic glycogen, leading to impaired synaptic plasticity [82] and depression-like behaviour [83].

## 3. Lactate Signalling in the Brain

**Figure 1:**
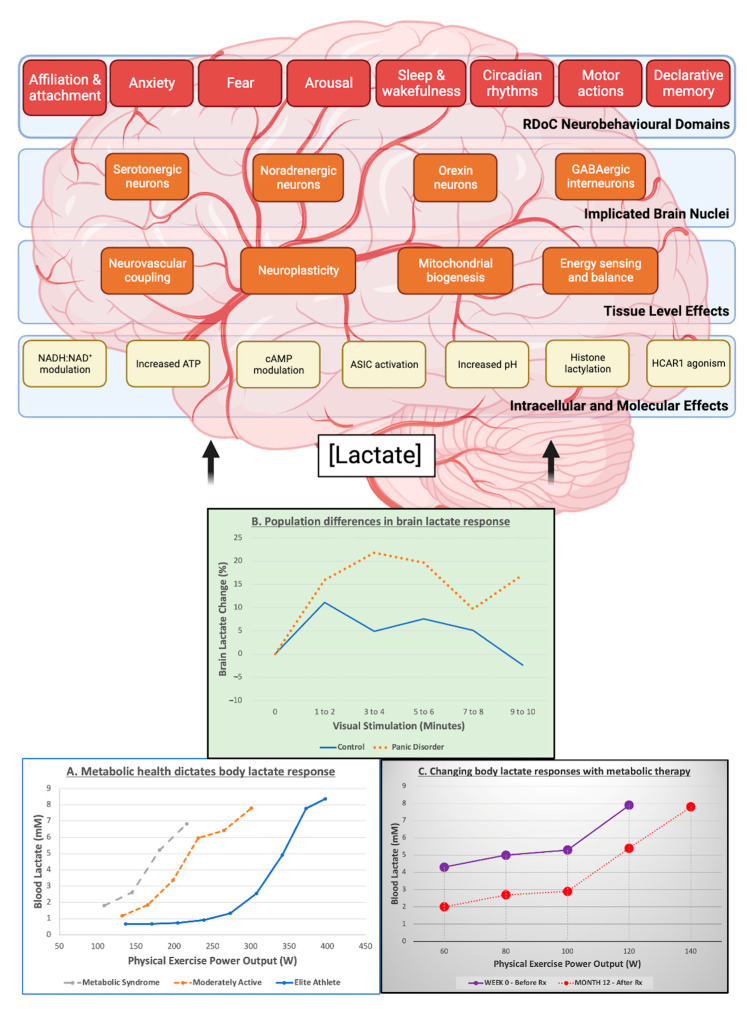
The implication of altered metabolic health on the molecular, intracellular, tissue level, and neurobehavioural effects of lactate in the brain. Metabolic syndrome (MS) is associated with mitochondrial dysfunction and impaired lactate oxidation. (**A**) In response to incremental exercise testing, participants with MS generate more circulating lactate at lower power outputs compared to individuals who do not have MS. Trained athletes who are metabolically fit can produce relatively less lactate at higher outputs. (**B**) In response to visual stimulus, participants with panic disorder produce more lactate in the visual cortex compared to a healthy control group. (**C**) In response to a personalized exercise prescription applied over 12 months, pre-type 2 diabetes was reversed, and the lactate response to incremental physical exercise was improved to reflect an improvement in mitochondrial function. Elevated concentrations of circulating or brain lactate can have effects on the brain, signalling at the molecular, cellular, tissue, and brain nuclei level to influence behaviour which map onto Research Domain Criteria (RDoC) domains. Figure inspired by [65], with graphical data adapted from [3,35,88].

When the systemic lactate concentration is elevated from baseline such as during strenuous physical activity the brain becomes a net consumer (above ~4 mM) [84,85,86]. The fate of this excess lactate within the brain is unclear, yet virtually every function of the brain can be influenced by it. It is important to highlight that depending on the metabolic profile of the individual, differential amounts of lactate may be produced and enter the brain over a time course which may have signalling implications [35]. Lactate displays mechanisms of action both extra- and intracellularly that are dependent on the intensity of the stimulus or level of activity, the balance between production and clearance by glycolysis and mitochondrial efficiency, the concentration gradient and diffusion properties of the tissue [87]. The molecular and intracellular mechanisms, tissue level effects, implicated groups of neurons, and RDoC-mapped neurobehavioural effects of lactate in the brain are described in the section below and summarised in Figure 1.

Extracellular lactate in the brain is associated with a relative reduction in pH [89]. Monocarboxylate transporters (MCTs), specifically MCT1-4, co-transport lactate with a proton, directly coupling lactate concentration and pH across compartments [90]. This intrinsic property of MCT1 on the blood–brain barrier therefore dictates that any lactate entering the cerebral compartment results in a relative reduction in pH. Generally, pH regulates synaptic transmission and neuronal firing via calcium voltage-gated channels and glutamate receptors [91]. Acid-sensing ion channels (ASICs) sense changes in pH and are distributed throughout the brain [92]. ASICs in the amygdala have been proposed as the target for lactate-induced panic [93]. Three other important brain areas also sense pH changes. The first is hypothalamic orexin neurons, a key energy sensing hub of the brain [94]. These neurons mediate autonomic arousal and influence wakefulness with widespread projections to major arousal-related centres including the thalamic nucleus, central grey, raphe nuclei, and locus coeruleus (LC) [95]. The second and third are serotonergic raphe nuclei [96] and the noradrenergic LC [97]. These nuclei have extensive projections that regulate arousal and neurovascular tone in the brain and body, and it almost goes without saying that both NE and serotonin are implicated broadly in mood and affective disorders.

Extracellular lactate can signal via membrane-bound receptors in the brain. In the brain, the hydroxycarboxylic acid receptor 1 (HCAR1) is found on plasma membranes of excitatory synapses within the hippocampus, cerebellum, and cortex [98]. It is also widely expressed by interneurons [99]. The activation of HCAR1 by lactate may decrease neuronal excitability and firing frequency [100], consistent with other data suggesting that lactate may act to suppress neuronal firing [101]. On the other hand, lactate appears to increase motor cortex excitability [102], and it may modulate high-frequency gamma oscillations [103]. These differences are likely related to temporal changes across distinct anatomical regions, with one group suggesting that activation of HCAR1 may aid memory consolidation after event-related activity [104]. Indeed, lactate can promote neurogenesis and angiogenesis via HCAR1 [105,106,107]. As of yet, no data link lactate receptors directly to mood and affective disorders, but HCAR1 does exert downstream effects on cyclic adenosine monophosphate (cAMP), and excessively elevated levels of cAMP are implicated in schizophrenia, fatigue, stress, cognitive impairment, and Alzheimer’s disease [99]. Other receptors for lactate are likely to exist, but so far remain elusive [108]. Perhaps most importantly, lactate directly stimulates LC neurons and can trigger an increase systemic blood pressure [109]. Even oral ingestion of lactate triggers the expression of tyrosine hydroxylase within LC neurons, the enzyme necessary for catecholamine synthesis [110]. Thus, extracellular lactate may have profound effects on neurocortical activity and arousal-related behaviours relevant to psychiatry. 

Brain lactate has important actions intracellularly where it maintains the redox state with pyruvate and the redox couple NAD^+^/NADH. In the brain, blood flow is modulated by this redox couple acting via a prostaglandin transporter that can respond to regional fluctuations in lactate [111] and enable efficient delivery of oxygen to areas that are most active [112]. Ca^2+^ is a key ion that can modulate mitochondrial metabolism in astrocytes [113], and similarly responds to the lactate-coupled NAD^+^/NADH ratio, modulating the astrocytic response to dopamine [114].

As a fuel of oxidative metabolism that can shuttle between mitochondria throughout the body, it would follow that a lactate sensor would exist in the brain to inform global energy balance and coupled behaviours. Indeed, the orexin neurons of the hypothalamus appear to act as the central lactate sensor, coupling the local concentration of lactate with their activity in an ATP-dependent manner [115]. 

Finally, great interest has been generated by the discovery of histone lactylation, a novel form of epigenetic modification [116]. Both extra- and intracellularly derived lactate driven by glycolysis or impaired mitochondrial function result in lysine lactylation and influence gene expression [117], leading to metabolic reprogramming [118]. One group found that neural excitation stimulated by social defeat stress in rodents precipitated H1 histone lactylation that, fascinatingly, correlated with a decrease in social behaviours [119]. Overall, these data highlight that lactate is a significant signalling molecule in the brain with pleiotropic effects relevant to the field of psychiatry.

## 4. Brain Lactate Dynamics as a Putative Therapeutic Biomarker

Chronic elevation of lactate raises ROS leading to mitochondrial dysfunction in a harmful feedback loop [120]. Thus, improving mitochondrial function and the associated lactate dynamics may potentially be useful for monitoring the metabolic effects of clinical therapy over the longer term. Paradigmatic examples of similar applications supporting this hypothesis are in the progressive neurodegenerative disorder Huntington’s disease, where elevated brain lactate could be reduced by Coenzyme Q10 (CoQ10) [121]. Similarly, in primary mitochondrial disorders where elevated lactate can be diagnostic, therapeutic approaches which target mitochondrial metabolism including CoQ10, riboflavin, and other B vitamins are trialled in order to improve mitochondrial function [122]. The notion that lactate might be a useful therapeutic biomarker in clinically defined populations such as those with metabolic syndrome is supported by evidence that measurements of peripheral lactate dynamics are sensitive to detect the effects of therapy associated with improvements in mitochondrial oxidative capacity, such as the reversal of a pre-type 2 diabetes through personalised exercise prescription [3,35]. The observations that systemic lactate is a proxy for mitochondrial efficiency that is sensitive to treatment effects, together with well documented evidence of mitochondrial brain abnormalities implicated in the aetiopathogenesis of psychiatric disorders, support our hypothesis that the ability to measure dynamic changes in lactate in the brain is likely to be a valuable avenue of research for psychiatric treatment. Data from interventional studies in psychiatric conditions, indicating that effective psychiatric treatments lead to brain lactate alterations, further reinforce this idea: in BPD patients, six weeks of lithium therapy was associated with a reduction in the concentration of lactate measured in the ACC to levels seen in healthy controls [123]. Twelve weeks of treatment with the anti-psychotic quetiapine correlated with a reduction in lactate in frontal areas of the brain, and responders exhibited greater decreases in brain lactate compared to non-responders [124]. Venlafaxine, a commonly prescribed antidepressant drug, was recently shown to have the ability to rescue metabolic changes in the hippocampus of rats exposed to chronic mild stress, raising the relative abundance of both glucose and lactate in cells compared to vehicle control [125]. In a small cohort of geriatric patients with BPD, just four weeks of CoQ10 supplementation improved depression scores measured with the Montgomery–Asberg Depression Rating [126]. With the knowledge that many psychiatric medications, in particular anti-psychotics, can impair or worsen metabolic health in patients, it is vital that old and new therapeutic approaches alike consider the metabolic effects.

### Therapeutic Interventions Targeting Brain Mitochondria and Lactate Dynamics

**Figure 2:**
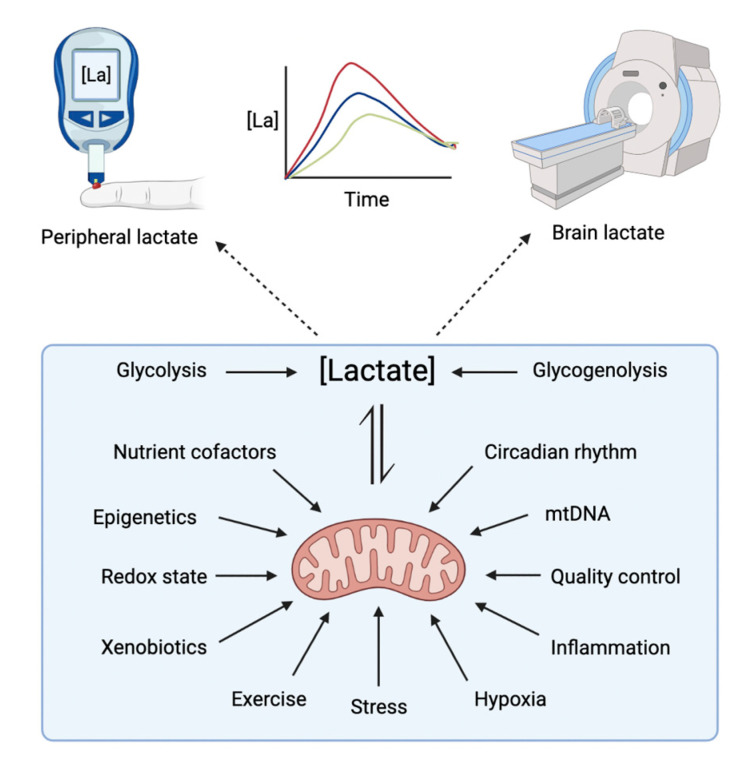
Therapeutic interventions that could influence lactate metabolism in the brain. Lactate concentration can be manipulated using metabolic therapies and subsequent changes measured with functional magnetic resonance spectroscopy (fMRS) in the brain or serum lactate in peripheral circulation (see dotted arrows). The concentration of lactate in the brain is primarily dependent on the rate of production influenced by the rates of glycolysis and glycogenolysis in the brain and body. On the other hand, the rate of lactate consumption relies on brain mitochondria. Mitochondria could be therapeutically manipulated in various ways (see solid arrows), and the outcome of interventions could be measured by observing changes in lactate dynamics.

In this section, we will discuss pathways and targets that could enable manipulation of lactate as a by-product of improving metabolism, most notably through improvements in mitochondrial oxidative efficiency. Many factors influence mitochondria, all of which present themselves as targets that could be leveraged for therapeutic benefit (Figure 2).

Physical exercise

It is well documented that physical exercise can have a positive impact on psychiatric symptoms. The physiological significance of lactate derived from exercise in the brain and the body is an active topic of discussion [127]. Just a single bout of high intensity exercise in mice corresponding to a blood lactate concentration of greater than 12 mM increased mtDNA copy number and the expression of Peroxisome proliferator-activated receptor Gamma Coactivator 1-alpha (PGC-1α) (the master regulator of mitochondrial biogenesis) in the hippocampus [128]. The inhibition of either MCT2 or the histone deacetylase (HDAC) Silent Information Regulator 1 (SIRT1) prevented this increase in hippocampal PGC-1α protein level, indicating that the observed effects are dependent on lactate transport acting via SIRT1 [129]. In contrast, low- or moderate-intensity exercise (3–4 mM) did not induce these changes in a single bout [128], consistent with data that repeated bouts are necessary at lower or moderate intensities [130]. In humans, low and moderate training intensity has been applied to specifically target mitochondrial function, improving plasma lactate and fatty acid clearance over the course of an individualised 12 month programme [3]. Strikingly, this approach has already been trialled as an adjuvant cancer therapy in order to target altered lactate metabolism, a metabolic hallmark of cancer [131]. With lactate metabolism in mind, the clinician has a new tool for tailoring the exercise prescription to alter and improve lactate dynamics on a case-by-case basis.

Pharmacological enhancement of mitochondrial function

The health of mitochondria in the brain is dependent on the processes that control their dynamics, number, and quality: mitophagy, biogenesis, fission, fusion, as well as the rate of mtDNA mutations [132]. Many compounds are under investigation for their beneficial effects on diverse clinical conditions and can target pathways that regulate mitochondria. Major large-scale randomised, placebo-controlled trials for neuropsychiatric conditions have already been called for by other groups due to the links between nitroxidative stress and brain bioenergetic failure, and compounds which have strong theoretical application with some convincing empirical data should be investigated further [133]. Key molecular nodes include SIRT1 and PGA-1α, which have been discussed above. Other important proteins that regulate mitochondria include the Peroxisome Proliferator-Activated Receptor (PPAR) transcription factors shown to regulate mitochondrial function in the brain [134]. Another is the conserved AMP-activated Protein Kinase (AMPK), which senses cellular energy status and can modulate several key elements of mitochondrial dynamics and quality [135]. Nuclear Factor erythroid 2-related Factor 2 (NRF2) is another transcription factor that coordinates the cellular antioxidant response but has been shown to broadly regulate mitochondrial quality control [136]. Several other less-specific targets are listed alongside a list of the most promising compounds known to cross the blood–brain barrier in Table 1. As a fundamental component of the electron transport chain, it is not surprising that Coenzyme Q10 has been shown to lower lactate levels in the brain in patients with neurological disorders [137]. Resveratrol, a polyphenol found in red grapes, significantly reduces the cerebral lactate accumulation in response to ischaemia in a mouse model of ischaemic brain injury [138]. Interestingly, metformin appears to have beneficial effects on a number of metabolic parameters but is documented to be an inhibitor of mitochondrial complex I and associated with lactic acidosis [139]. Overall, we feel that in measuring the dynamic lactate response to these pharmaceutical agents, and doing so over time, will yield valuable information that enables precise and personalised approaches to metabolic psychiatry.

Nutritional and dietary intervention

Alongside the micronutrients outlined in Table 1, dietary practices that include intermittent fasting (IF), calorie restriction (CR), and the ketogenic diet (KD) are purported to have an impact on mitochondrial function and quality control. Both IF and CR alter mitochondrial dynamics and improve biogenesis and ROS production [221]. Long-term CR in rats reduces the accumulation of liver mtDNA oxidative damage as a result of altered ROS production [222]. Fasting-induced ketosis acutely increases brain lactate [223], which does not occur with infusion of exogenous β-hydroxybutyrate [224], thus being a phenomenon speculated to be underpinned by the chronic adaptive upregulation of brain MCTs. Indeed, the anti-convulsant effects of the KD and induced mitochondrial biogenesis may only emerge after weeks of epigenetic adaptation [225]. The proposed beneficial effects of the KD may be mediated by a shift in the NAD^+^/NADH ratio [226], giving insight into how it could modulate lactate concentration. Further understanding the dynamic brain lactate response acutely and over time to any proposed nutritional or dietary protocol may provide valuable insight that explains how these approaches alter brain neurometabolism with relevance for psychiatric disorders.

Stress, hypoxia, and inflammation

Oxidative stress and inflammation impair mitochondrial function and result in excessive ROS production [227]. Acute and intermittent lactate exposure is adaptive [228] and can promote oxidative stress resistance, upregulating cellular defence mechanisms via NRF2 [229]. However, chronic exposure to lactate alters fatty acid metabolism and membrane composition, increasing ROS production and decreasing ATP production in mouse cardiomyocytes, resulting in further impairment of mitochondrial function [120]. Hypoxic and pseudohypoxic (NAD^+^ depleted) states lead to the stabilisation of Hypoxia Inducible Factor 1-alpha (HIF1-α) downstream of SIRT1 that disrupts mitochondrial function through genome desynchrony [230]. It almost goes without saying that hyperbaric oxygen therapy (HbO_2_) leads to a reduction in lactate concentration in skeletal muscle [231]. Cyanide leads to an accumulation of lactate in the rat brain that can be measured with microdialysis and ameliorated with HbO_2_. On the other hand, some evidence suggests that acute hypoxia may transiently improve metabolic parameters [232], and the lactate response to this may be an important variable to consider. HbO_2_ or acute hypoxia may represent novel treatment avenues for mood and affective disorders that deserve investigation tailored to the individual based on changes in lactate metabolism.

Circadian rhythm

Circadian biology is a key consideration for improving the health of mitochondria. This relationship is biochemically underpinned by melatonin, a mitochondrially synthesised hormone controlled by light that enhances electron transport, ATP synthesis, and ROS [233]. Knowing that lactate is associated with the sleep–wake cycle, and that a chronic elevation of lactate can result in excessive ROS production and mitochondrial dysfunction [120], addressing this axis with patients is then critical. This link enables the clinician to go beyond classical sleep hygiene measures and towards metabolic therapy for improving conditions such as insomnia.

Other examples of lactate manipulation

Lactate can be elevated via intravenous infusion of sodium lactate solution, documented to exhibit panicogenic and anti-depressive effects [42,66]. In a model of SCZ, exogenous lactate rescued the abnormal behaviour of DISC transgenic mice [234]. Early work discovered that repeated infusion led to a reduction in the anxiogenic response in participants over time, likely reflecting habituation to the interoceptive signals associated with elevated lactate [235]. Electroconvulsive therapy (ECT) is currently employed in psychiatry, and seizure activity is associated with elevations in circulating lactate and is elevated above 4 mM in those who suffer with post-ECT agitation but not in those who do not [236]. Whole-body hyperthermia has similar effects [237]. Several psychiatric medications influence the catecholamine system through their mechanism of action, and therefore, one would expect this to have an impact on lactate production, a worthwhile consideration for future work.

## 5. Brain Lactate Measurement with Functional Magnetic Resonance Spectroscopy

Proton magnetic resonance spectroscopy (^1^H-MRS) can detect and quantify lactate in brain tissue without administration of an exogenous tracer. Lactate exhibits a distinct peak with a chemical shift around 1.3 parts per million (ppm) within the spectra. This well-separated position from other metabolite resonances appears as a doublet due to the coupling between its two proton environments that can reflect the biochemical or biophysical properties in the environment. 

The in vivo detection of lactate with ^1^H-MRS yields challenges. The lactate resonance has a relatively low signal strength compared to other metabolites and spectral overlap from compounds such as lipids and other macromolecules require careful analysis and spectral fitting to distinguish and quantify it. Shimming reduces inhomogeneity in magnetic fields and ensures that lactate appears at the expected 1.3 ppm spectral position. Various acquisition techniques can be employed, with the choice of echo time (TE) playing a crucial role. Short TE approaches are used to detect lactate minimizing the decrease in signal due to T_2_ relaxation, although these may increase the contribution from macromolecules. Long TE approaches reduce contributions from broad resonances but may decrease sensitivity to lactate. J-editing is an approach that selectively detects lactate by exploiting scalar coupling between the three protons on the C2 position and the proton on the C1 position in the lactate molecule. This approach suppresses unwanted signals and thereby enhances the lactate resonance. The drawback of lactate editing is a 40% reduction in SNR, compared to a non-edited acquisition with the same number of spectral averages. The choice of acquisition parameters, including TE, spectral editing techniques, and pulse sequences, is likely to depend on the MR field strength. It goes without saying that higher field strengths provide increased SNR and improved spectral separation, thus enhancing lactate detection.

Early work identified that photic stimulation of the visual cortex precipitates a rise in lactate concentration to 0.3–0.9 mM [74]. The visual stimulation paradigm effectively enables the quantification of lactate and other metabolites in the visual cortex due to high energy metabolism and proximity of the surface coil. Notably, it is in the visual cortex that differential lactate responses in panic disorder participants have been observed [88]. Other groups have been able to monitor a range of energy metabolites including lactate, ATP, and phosphocreatine (PCr) levels, shedding light on the metabolic demands of the motor cortex during movement [238]. However, although robust and replicated evidence reported altered lactate levels in areas of the brain relevant to mood and affective disorders, such as the anterior cingulate cortex, very few studies have aimed to quantify dynamic lactate responses in those areas with functional task-based paradigms [239]. 

In animal studies, microdialysis can be employed as a complementary technique to validate the MRS-derived signals [240]. This technique is challenging for many reasons, particularly because lactate responds to anaesthesia or immobilisation and other forms of stress, further highlighting the nature of lactate as a moment-to-moment biomarker of neuroenergetics.

## 6. Conclusions

A growing body of evidence already highlights the importance of cerebral lactate measurement in mood and affective disorders. Recent meta-analyses of both BPD and SCZ have identified alterations in brain lactate [31,241]. BPD in particular is strongly associated with evidence of mitochondrial dysfunction [32], and it appears that brain lactate can be modified by current pharmacological therapies in these patients [123,124]. We hope that by placing these data within a framework that enables a thorough understanding of their nuance, we can drive the development of better technology and research paradigms required to make precise and useful measurements [242,243]. 

In the present review, we have outlined roles for lactate that map onto each domain of the National Institutes of Mental Health RDoC framework (Figure 1) [34]. With data from both rodent and human studies, lactate in the brain is panicogenic [244] and anti-depressive [42], involved in social threat perception [245], memory formation and executive function [80,246], tied to circadian rhythms and arousal [62,78], and reflects motor activity [102]. Simple questions we can ask for future work relating to the field of metabolic psychiatry would be as follows: Where and when is lactate elevated? How long does it persist? And why?

Any therapeutic intervention should have both objective and subjective outcomes that allow a clinician and patient to monitor and validate progress. If we reconsider the seminal observations of abnormal lactate responses to exercise in anxious populations from our updated metabolic perspective, then we should see this as an opportunity for intervention that can be personalised and monitored over time. This has implications for drug development, mitochondrially targeted nutraceuticals or dietary interventions, and for developing exercise protocols that can modulate the capacity to produce or use lactate, influencing the temporal dynamics. 

Overall, we posit that lactate connects metabolic stress at the cellular level to stress at the macroscopic level and, as such, naturally presents itself as a theranostic biomarker for furthering the field of metabolic psychiatry.

## Figures and Tables

**Table 1:** Compounds that target mitochondrial function known to penetrate the CNS.

Compound	Target	Mechanism	Clinical Trials	References
Bezafibrate	PPARα	↓Fission ↑Mitophagy ↑Biogenesis	Primary Biliary Cirrhosis Cardiovascular Disease	[140,141,142,143]
Ciclopirox olamine	PGC-1α Metal Chelator	↑Mitophagy ↑Biogenesis	Anti-tumour	[144,145,146]
Coenzyme Q10	Electron Acceptor	↑Mitophagy	Huntington’s Disease Parkinson’s Disease	[147,148,149]
Deferiprone	Iron Chelator	↑Mitophagy	Parkinson’s Disease Friedrich’s Ataxia Alzheimer’s Disease HIV Thalassaemia Sickle Cell Disease	[150,151,152,153,154,155]
Elamipretide	Cardiolipin Stabiliser	↓Fission ↑Fusion ↑Mitophagy ↑Biogenesis	1° Mitochondrial Myopathy Macular Degeneration Myocardial Infarction Heart Failure	[156,157,158,159,160,161]
Fisetin	Antioxidant SIRT1/AMPK PPAR**γ**	↓Fission ↑Fusion ↑Mitophagy ↑Biogenesis	COVID-19 Cancer Stroke	[162,163,164,165,166,167]
Fluvastatin	HMG-CoA Reductase	↑Mitophagy ↑Biogenesis	Systemic lupus erythematosus Metabolic syndrome Hepatitis C	[168,169,170,171,172,173]
Luteolin	Flavanoid Multiple Targets	↓Fission ↑Fusion ↑Mitophagy ↑Biogenesis	COVID-1916/08/2023 18:44:00 Autism	[174,175,176,177]
Metformin	AMPK	↓Fission ↑Fusion ↑Mitophagy ↑Biogenesis	Schizophrenia Bipolar Affective Disorder Migraine Stroke	[178,179,180,181,182,183]
Methylene Blue	NRF2 Electron Recycling	↓Fission ↑Fusion ↑Mitophagy ↑Biogenesis	Post-traumatic Stress Disorder Bipolar Affective Disorder Alzheimer’s Disease Post-operative Delirium Frontotemporal Dementia	[184,185,186,187,188,189,190]
Nicotinamide	SIRT1	↓Fission ↑Fusion ↑Mitophagy ↑Biogenesis	Friedrich’s Ataxia Skin Cancer Diabetes Parkinson’s Disease	[191,192,193,194,195]
Oxaloacetate	Antioxidant TCA cycle SIRT1	↑Biogenesis	COVID-19 Chronic Fatigue Syndrome Parkinson’s Disease Premenstrual Syndrome	[196,197,198,199]
Piracetam	NRF2	↓Fission ↑Fusion ↑Biogenesis	Stroke Dementia Sickle Cell Disease Foetal Distress in Labour	[200,201,202,203,204,205]
Pterostilbene	PI3K-Akt-mTOR SIRT1	↑Mitophagy ↑Biogenesis	Infertility Muscle Injury Amyotrophic Lateral Sclerosis Acute Kidney Injury	[206,207,208,209,210,211]
Resveratrol	PPARs	↓Fission ↓↑Fusion ↑Mitophagy ↑Biogenesis	Diabetes Schizophrenia Alzheimer’s Disease COVID-19	[212,213,214,215,216]
Sulforaphane	NRF2	↓Fission ↑Fusion ↑Mitophagy ↑Biogenesis	Autism Depression Schizophrenia	[217,218,219,220]
